# Risk Stratification of COVID-19 Using Routine Laboratory Tests: A Machine Learning Approach

**DOI:** 10.3390/idr14060090

**Published:** 2022-11-21

**Authors:** Farai Mlambo, Cyril Chironda, Jaya George

**Affiliations:** 1 School of Statistics and Actuarial Science, University of the Witwatersrand, 1 Jan Smuts Ave, Braamfontein, Johannesburg 2000, South Africa; 2 Department of Chemical Pathology, University of Witwatersrand, 29 Princess of Wales Terrace, Parktown, Johannesburg 2193, South Africa; 3 National Health Laboratory Services of South Africa, 1 Modderfontein Road, Sandringham, Johannesburg 2131, South Africa

**Keywords:** COVID-19, machine learning, risk stratification, laboratory tests

## Abstract

The COVID-19 pandemic placed significant stress on an already overburdened health system. The diagnosis was based on detection of a positive RT-PCR test, which may be delayed when there is peak demand for testing. Rapid risk stratification of high-risk patients allows for the prioritization of resources for patient care. The study aims were to classify patients as severe or not severe based on outcomes using machine learning on routine laboratory tests. Data were extracted for all individuals who had at least one SARS-CoV-2 PCR test conducted via the NHLS between the periods of 1 March 2020 to 7 July 2020. Exclusion criteria: those 18 years, and those with indeterminate PCR tests. Results for 15437 patients (3301 positive and 12,136 negative) were used to fit six machine learning models, namely the logistic regression (LR) (the base model), decision trees (DT), random forest (RF), extreme gradient boosting (XGB), convolutional neural network (CNN) and self-normalising neural network (SNN). Model development was carried out by splitting the data into training and testing set of a ratio 70:30, together with a 10-fold cross-validation re-sampling technique. For risk stratification, admission to high care or ICU was the outcome for severe disease. Performance of the models varied: sensitivity was best for RF at 75% and accuracy of 75% for CNN. The area under the curve ranged from 57% for CNN to 75% for RF. RF and SNN were the best-performing models. Machine Learning (ML) can be incorporated into the laboratory information system and offers promise for early identification and risk stratification of COVID-19 patients, particularly in areas of resource-poor settings.

## 1. Introduction

With the emergence of COVID-19 caused by the novel SARS-CoV-2 [1], in late 2019 and the early months of the year 2020, the world was put on hold. Since 17 January 2020, when the World Health Organization (WHO) announced it as an international public health concern, the numbers of people who contracted COVID-19 increased dramatically. As of 15 November 2022, the confirmed cases stood at 635 million with a death toll of 6.6 million people worldwide. These figures are four million and 102 thousand respectively, for South Africa (https://www.google.com/search?q=covid+19+world+stats&rlz=1C1VDKB_enZA943ZA944&oq=&aqs=chrome.0.69i59i450l8.107752819j0j15&sourceid=chrome&ie=UTF-8, accessed on 20 September 2022). These numbers led the world leaders to seek solutions to contain viral spread. Among many methods, national lockdowns, social distancing, and wearing protective face masks were used and these methods proved to be effective in at least the management of the viral spread [2]. South Africa was in various national lockdown levels from April 2020 lasting until June 2022.

The rapid spread, high infectivity and quick progression of the disease in positive patients [3] stressed the health care systems. This stress meant that there was always an urgent need for quick and effective diagnostic and risk stratification measures, in order to identify patients that require intensive care. The most common test for the virus is the PCRs which involves testing swabs from various respiratory tracts, but mainly the nasopharyngeal swab [4]. Delays in the traditional risk stratification methods causes a lag in hospital admission time and bed assignment for patients as well as possible exposure of the healthcare givers to infected patients.

This study aimed at using machine and statistical learning models to predict the severity of COVID-19 using quick and readily accessible routine laboratory tests. The problem is approached as a classification problem using supervised, machine learning algorithims. Routine lab tests are available within 30 min to 2 h. The tests results provide a number of analyte markers values which, together with some pre-known health conditions, can be used for risk stratification. An analyte is a biochemical compound, which is a target for chemical analysis, hence an analyte marker is made by spiking the compound to make it effective in measurements. Artificial intelligence has inspired machine learning algorithms capable of the initial diagnosis and risk stratification of various diseases [5,6,7,8,9,10,11].

### 1.1. Aim

The aim of the research was to classify whether the positive COVID-19 cases had severe COVID-19 symptoms or not, hence helping to decide the type of hospital ward where the patients would be admitted. Severe COVID-19 symptoms include: difficult breathing, body weakness, high fever and muscle and joint pains. The classification was carried out using the data provided from a network of laboratories in South Africa, which contains various analyte measures. The analytes were used as variables in the classification.

### 1.2. Objectives

The objectives followed the aim. These were:

- To use machine learning and statistical learning models to classify the severity of COVID-19, which is (RS).
- To compare the fitted machine learning models using different measures of performance.
- To identify the top-performing model for the above-mentioned objectives.
- To identify the top important analytes relevant to the risk stratification of COVID-19.

### 1.3. Research Design

A retrospective study design was used to describe results extracted from the Central Data Warehouse (CDW) of the National Laboratory Health Services (NHLS), for all patients tested between 1 March 2020 and 7 July 2020, in the public sector healthcare facilities of the country. The CDW houses all laboratory results for public sector patients in South Africa.

### 1.4. Data

We extracted data for all individuals who had at least one PCR test conducted via the NHLS between 1 March 2020 and 7 July 2020. Patient data was anonymized to prevent traceablility. A six month period of demographic, biochemical and haematological and microbiology data was extracted for all patients who had a SARS-CoV-2 PCR test. Out of a total of 842,197 tests, 11.7% were positive and 88.3% negative. A critical case was defined as a patient who was admitted into a ward because of COVID-19 complications and non-critical patients are positive cases that were not admitted.

## 2. Literature Review

This section presents a review of the findings of various studies that are similar to this study. The literature review goes through various papers, and other publications that looked, directly or indirectly to the use of machine learning in prediction of COVID-19 as well as risk stratification. Multiple articles were written and/or presented that used different predictors, which include analytes and imaging techniques to predict and stratify COVID-19 risk. The section analyses and critiques various methods used by other researchers as well.

### 2.1. Background of Using Routine Lab Results for COVID-19 Diagnosis

**Figure 1:**
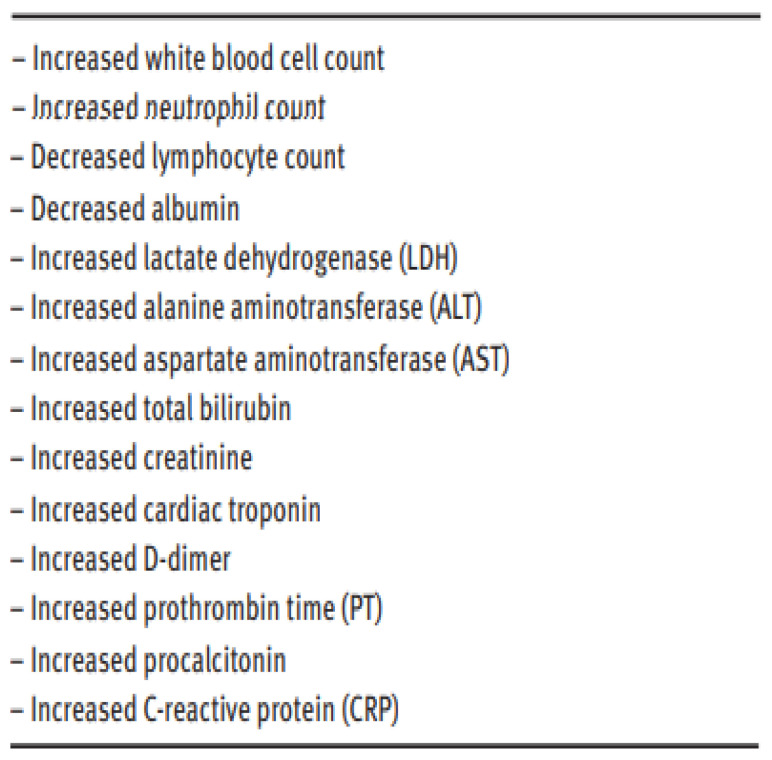
Main abnormalities noted by [12] in COVID-19 patients.

Clinical characteristics of COVID-19 have been well studied and a lot of abnormalities have been noticed in patients infected with the disease [12]. These abnormalities demonstrate that they play an important role in early diagnosis, detection and even management of the disease [13]. The table in Figure 1 shows the findings of their research and how COVID-19 affected the various listed analytes.

With the devastating nature of COVID-19, it is important to identify and rank groups of patients who are at risk of severe COVID. Several authors have documented risk factors mostly associated with severe COVID-19 outcomes. These studies included mostly comorbidity conditions such as HIV infection [14], type 1 and type 2 diabetes [15]. Hesse et al. [16] presented findings using the same data as used for this research. The study looked at how comorbidity factors which include: HIV, TB, and Diabetes HBA1c and other related laboratory analytes affect the severity of COVID-19. All these studies demonstrated that it is possible to use routine lab test results and comorbidity factors to predict and diagnose COVID-19 status and severity.

### 2.2. Machine and Statistical Learning in Predicting COVID-19 Severity

Zimmerman et al. [17] review the prospective uses of machine learning and artificial intelligence for cardiovascular diagnosis, prognosis, and treatment in COVID-19 infection in a number of cardiovascular applications. Applications of Artificial intelligence, particularly machine learning, have the potential to take advantage of platforms with a lot of data and change how cardiovascular illness is identified, risk-stratified, prevented, and treated. The authors also cite how improvements in AI have been made in various fields of cardiology.

There have been various studies that have documented how statistical and machine learning models can be used to diagnose and predict COVID-19 and its severity [18,19,20]. These have used supervised learning albeit with different features. The models that have been developed demonstrated great predictive performances.

**Figure 2:**
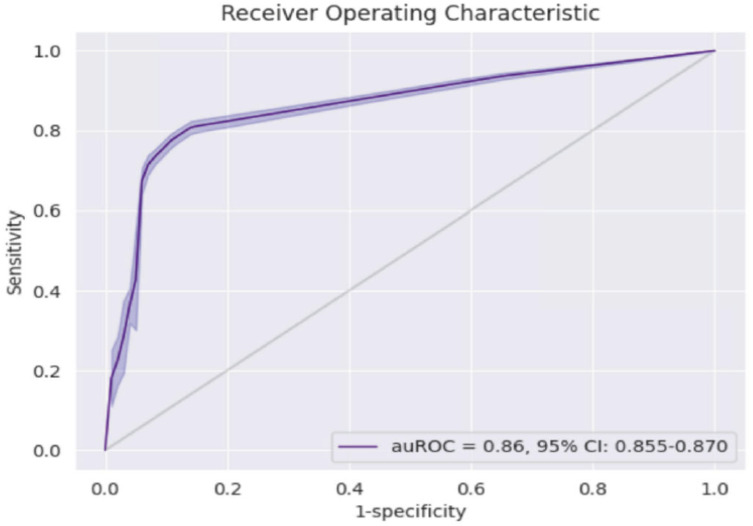
ROC curve showing model performance with high AUC of 86% [18].

Zoabi et al. [18] used machine learning models to diagnose COVID-19 based on symptoms experienced. The study features space comprised of sex, age, and symptoms such as cough, fever, sore throat, shortness of breath, headache, and whether one was in contact with a positive COVID-19 case. The study employed the gradient-boosting model in Python and used the ROC curve to assess model performance with the bootstrap re-sampling method. The model demonstrated high predictive performance with the area under ROC curve being 86% as shown in Figure 2.

**Figure 3:**
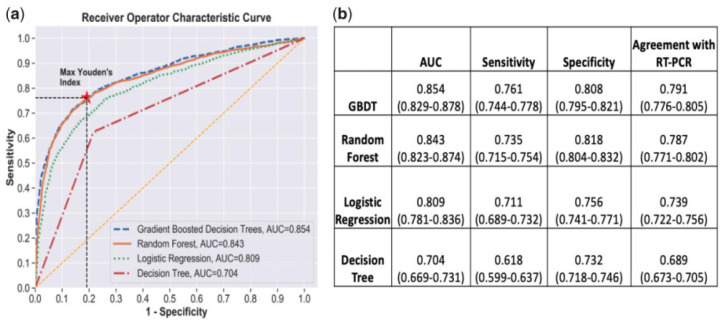
5-Fold Cross-validation of the four models stated. (**a**) Comparison of ROC curves (**b**) comparison of AUC, specificity, and sensitivity. Gradient Boosting DT is shown to be the best model [19].

Yang et al. [19] used 27 laboratory tests together with demographic features (age, sex, race) to fit machine learning models in the R software [21] for COVID-19 prediction and risk stratification. The research fitted a logistic regression classifier, decision tree, random forest, and gradient-boosting decision tree classifiers. A 5-fold cross-validation re-sampling method was employed, with the area under the ROC curve used predominantly as the measure of model performance. As shown in Figure 3, the two ensemble models which are gradient boosting models and random forest outperformed the singular models of logistic regression and single decision tree in that order. All the models, however, had a high predictive performance.

**Figure 4:**
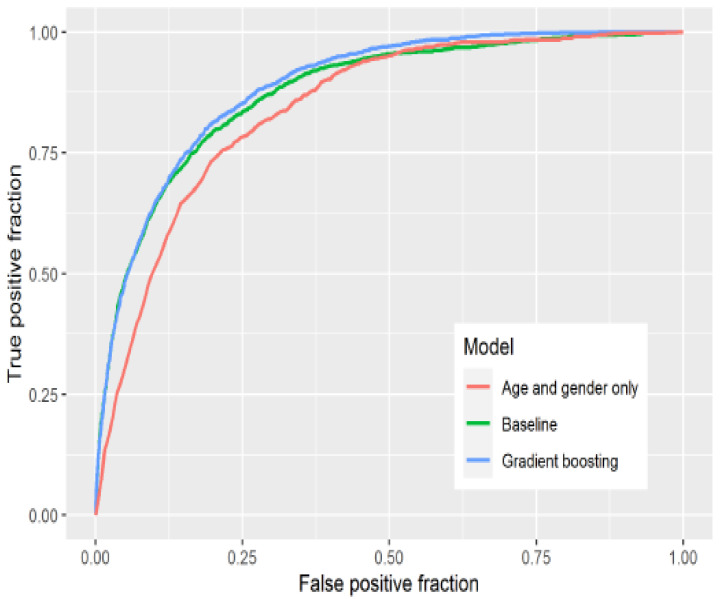
Adapted from [20], the figure shows a set of ROC curves for gradient boosting and logistic regression models.

Jucknewitz et al. [20] used statistical learning to analyse prior risk factors for the severity of COVID-19. The study used factors such as age, gender, nationality, occupation, employment, income, etc. LASSO was used in variable selection, and a regression model together with gradient boosting models were used. ROC, AUC, and accuracy were used to evaluate the models’ performances. Figure 4 shows the ROC curves for the models, the gradient boosting model shows an AUC of 88.79%, and the baseline model had an AUC of 87.55%.

**Figure 5:**
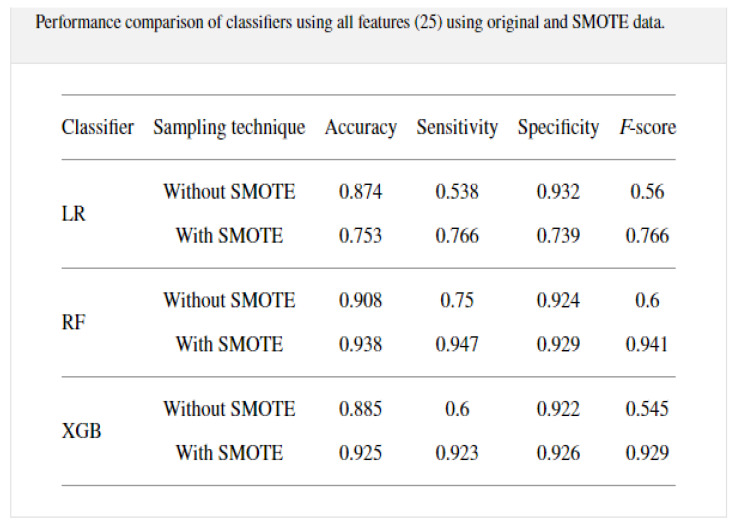
Table of results adapted from [22] of the study from King Faha University Hospital in Saudi Arabia, using the three machine learning models.

A study was conducted from 287 COVID-19 samples from King Faha University Hospital in Saudi Arabia by [22] on prediction of the disease using three classification algorithms, namely, random forest, logistic regression, and extreme gradient boosting model. The data was re-sampled using 10-k cross-validation with SMOTE to alleviate the imbalances that were present. The modeling was conducted on 20 features that included some symptoms as well. The RF model outperformed the other classifiers with an accuracy of 0.938, sensitivity of 0.947, and specificity of 0.929, with the results given in a table shown in Figure 5.

Alballa et al. [23] compiled a review of a number of studies that employed machine learning in COVID-19 diagnosis, mortality and risk predictions. The study noticed that most studies employed supervised machine learning models. The papers aims were to:

- Review ML algorithms used in the field mainly used for diagnosis of COVID-19 and prediction of mortality risk and severity, using routine clinical and laboratory data that can be accessed within an hour.
- Analyses the top features/variables that were found to be top predictors, i.e., the most important features relevant to machine learning predictor models.
- Outline the algorithms mostly used and for which purpose.
- Points out some areas of improvement as well as areas of further study.

**Figure 6:**
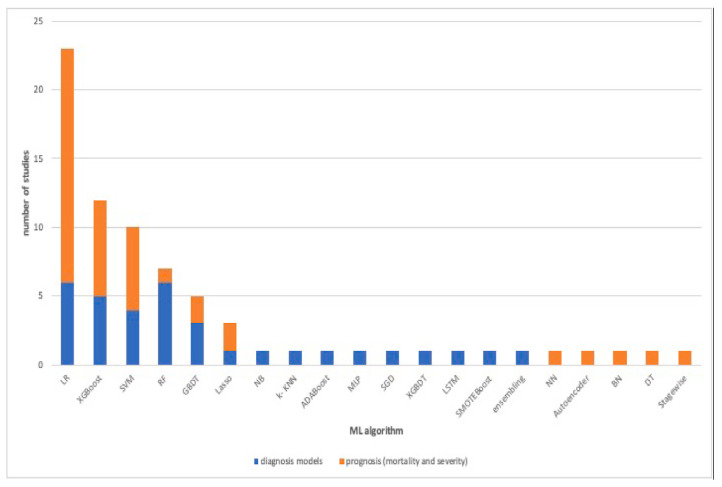
The machine and statistical models used for COVID-19 diagnosis and prognosis adapted from [23].

**Figure 7:**
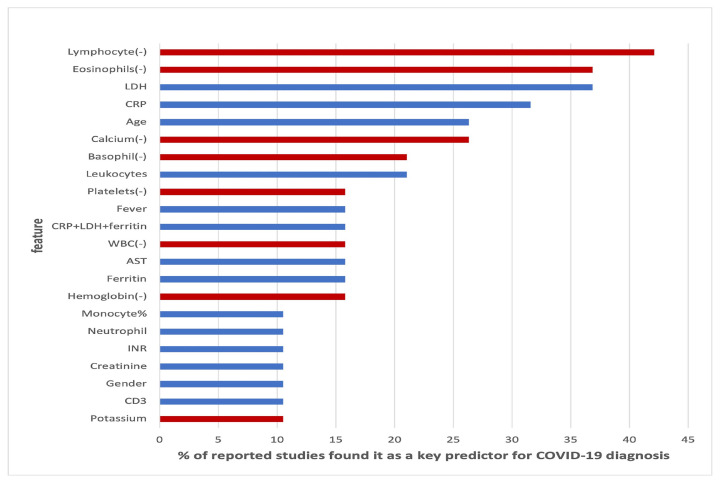
Top features found as most important features in diagnosis of COVID-19 modified from [23].

**Figure 8:**
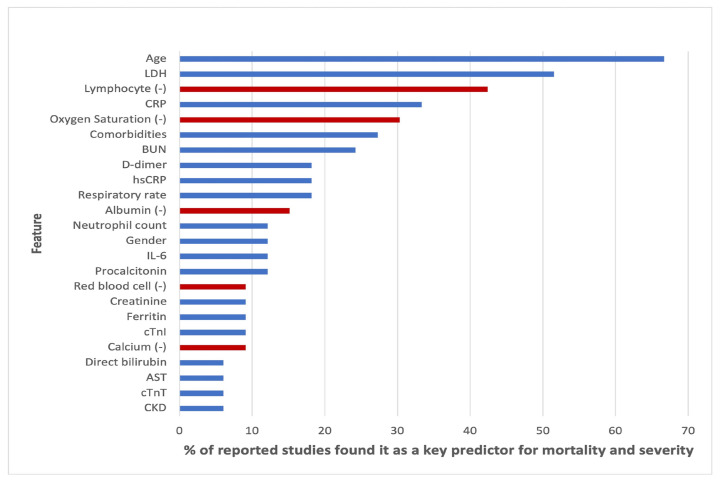
Top features found as most important features in prognosis of COVID-19 modified from [23].

The paper concluded that the results of machine learning and statistical models are consistent with those of pure medical studies. It also pointed out the issue of imbalance and missing values in the data usually used in the studies. The results from their study are shown in Figure 6, Figure 7 and Figure 8.

## 3. Methodology

This section provides detailed explanations and descriptions of various methods that were implemented and used in the study in order to arrive at the intended results.

Consider supervised data with predicted variable Y and predictor variables $X=(X_{1},X_{2},\cdots ,X_{p})$, $\bar{X}$ is a vector representation of the predictor variables $X_{i}$, i.e., the predictor variables and analytes from routine clinical tests. Let $Y_{i}\in \left\{0,1\right\}$ be an indicator variable with $Y_{i}=0$ be not-Severe COVID-19 and $Y_{i}=1$ be Severe COVID-19. This study classified this supervised data set for Logistic regression, Decision Trees, Random Forest, Extreme Gradient Boosting, the Self Normalising Neural Network and the Convolutional Neural Network.

### 3.1. Missing Values

The data used in this study contained missing values. The missingness of values in the data is both structural missing (data missing because of an explainable reason, e.g., patients who did not get any blood tests because they were not hospitalised) and Missing Completely at Random (MCR) (i.e., data missing because of reasons that cannot be traced). This is because some patients were not admitted or some test values were not available.

#### 3.1.1. missForest Missing Value Imputation

missForest is a non-parametric missing value imputation method that uses the random forest algorithm [24] on every single variable to estimate and predict the missing values. We used the package missForest [25] in R which enables control of the process with an adjustable number of trees, number of iterations and other parameters to tune.

#### 3.1.2. Simple Statistics Missing Value Imputation (SSMVI)

SSMVI is a non-parametric method of missing values imputation which assumes a symmetric distribution of the data points of any given variable [26,27]. This imputes numeric missing values with the mean of the observed values and imputes factor values with the modal class of the observed values. We created an algorithm that implemented what is known as a predictive mean matching for numeric variables in R, as well as predictive mode matching for factor variables.

### 3.2. Variable Selection

#### 3.2.1. Boruta Algorithm for Feature (Variable) Selection

With the high volumes of data presented in the machine and statistical learning modeling practices, it is of much necessity to reduce the volume of the data, particularly the number of variables. This process is conducted by removing redundant and correlated features, which in turn helps to produce non-complex models that are relatively easy to interpret and faster to compute [28].

The Boruta algorithm was named after the Slavic mythology god of the forest, as it modifies and improves on much of the variable importance algorithm used in RF models [29,30] (Algorithm 1).
**Algorithm 1:** Boruta Variable Selection Algorithm [29]Create Shadow Features: the data set is duplicated column by column and all values are randomly permuted and hence removing any relationship that might have originally existed.Random Forest Training: the data set is trained using a random forest classifier and the variable importance from the training are collected.Comparison: for each variable, the algorithm compares the feature importance of the original variable and the maximum importance of all shadow variables (The best shadow variable). A shadow variable is one that has been created with similar characteristics as the original variable given in a data set. If the feature importance is higher than the best shadow variable it is recorded as an important variable.Iterations: the process continues until a pre-defined number of iterations is obtained and a table of hits is recorded and these are the variables that will be selected for the model.

The Boruta algorithm is widely used as it gives the user more flexibility in the number of iterations one can run and has produced good results for biomedical data [30]. We used the Boruta package [29] in R, which is computationally cheap and gave the advantage of tuning the number of trees and the number of iterations.

#### 3.2.2. LASSO Feature Selection

The method was first coined by [31]. The Least Absolute Shrinkage and Selection Operator (LASSO) concentrates on doing two fundamental tasks, i.e., regularisation and feature selection, with regularisation being the driving factor used in the feature selection. Regularisation is defined as the reduction of data values towards a central point, usually the mean. LASSO introduces a penalty over the sum of the absolute values of the coefficients of the model (model parameters). This results in shrinking (regularisation), where some of the coefficients are shrunk to zero. In the feature selection process, the variables that will remain with a non-zero coefficient value (after shrinking) are then selected for modeling. This is conducted with the objective of minimising the prediction error (SSE) [32].

The strength of the penalty is controlled and determined by the value of a tuning parameter say ζ. The larger the value of ζ, the more the coefficients are forced to zero, hence more variables are rendered insufficient during shrinking. Notice that if ζ=0, the model is an OLSs regression [31,32].

The study used the Buhlmann and Van de Geer formulation of the LASSO modeling [33], for a linear model

(1) $$Y=\bar{X}\beta +\epsilon$$

with $\bar{X}$ and Y as vectors, defined before, with β being the the coefficient matrix and ϵ being the error vector. The LASSO estimate is defined by the solution to the $l_{1}$ penalty optimisation problem.

(2) $$\mathrm{minimise}\left(\frac{||Y-\bar{X}\beta {\left|\right|}_{2}^{2}}{n}\right)\mathrm{subject}\;\mathrm{to}\sum_{}^{p}{\left||\beta |\right|}_{1}<t$$

where *t* is defined as the upper bound of the sum of all coefficients $\beta_{i}$ for *n* data points. This minimisation is the same as the parameter estimation that comes after

(3) $$\beta \left(\zeta \right)=\underset{\beta }{\mathrm{argmin}}\left(\frac{||Y-\bar{X}\beta {\left|\right|}_{2}^{2}}{n}+{\zeta ||\beta ||}_{1}\right)$$

where $||Y-\bar{X}{\beta ||}_{2}^{2}=\sum_{i=0}^{n}(Y_{i}-{\left(\bar{X}\beta_{i}\right)}^{2}{,||\beta ||}_{1}=\sum_{j=1}^{n}\left|\beta_{j}\right|$ and ζ≥0 is the penalty parameter.

Lasso was used in conjunction and compared to Boruta. LASSO gives accurate models, since the shrinking process results in reduced bias. Model interpretability is highly improved by LASSO due to the elimination of irrelevant features [32,33]. The study used the Caret package [34] to perform LASSO in R as it allows adjustments of various parameters and is computationally cheap.

### 3.3. Logistic Regression

Let π indicate the probability of a patient being COVID-19 positive, and let $\beta_{i}$ be regression coefficients associated with the feature $x_{i}$ and $\beta_{0}$ be the intercept. Presently, a logistic regression (LR) model [35] is given by the equation:

(4) $$\log\left(\frac{\pi_{i}}{1-\pi_{i}}\right)=\beta_{0}+\sum_{i=1}^{n}\beta_{i}\left(x_{i}\right)$$

Regression coefficients can be fitted using the maximum likelihood estimation [36]. To solve each probability of success using the logit use:

$$\pi_{i}=\frac{\exp\left(\beta_{0}+\sum_{i=1}^{n}\beta_{i}\left(x_{i}\right)\right)}{1+\exp\left(\beta_{0}+\sum_{i=1}^{n}\beta_{i}\left(x_{i}\right)\right)}$$

The probability can be estimated using a set threshold [36,37] to determine which class a patient belongs to. The study used a threshold of 0.5, that is, if $\pi_{i}>0.5$, then a patient belongs to the positive class and if $\pi_{i}\leq 0$, then the patient belongs to the negative class. Based on the algorithm, predictions are made to quantify the accuracy and other measures of performance. We used the Caret package [34] in R to fit a logistic regression. The package allows the adjustment of various parameters as well as the implementation of automatic cross-validation.

### 3.4. Tree-Based Methods

#### 3.4.1. Decision Tree

Decision Tree (DT) algorithms are non-parametric techniques that seek to classify data according to various rules where they continuously divide and split (divide and conquer) the feature space [38,39]. The partitioning splits the feature space into small chunks of non-overlapping spaces, whose response values correspond as guided by the set rules. The predictions are then obtained by fitting simple models (such as a constant) to each chunk of space [40]. DT are used, since they require less assumptions compared to classical methods and can handle huge varieties and types of data [39].

##### Tree Structure

**Figure 9:**
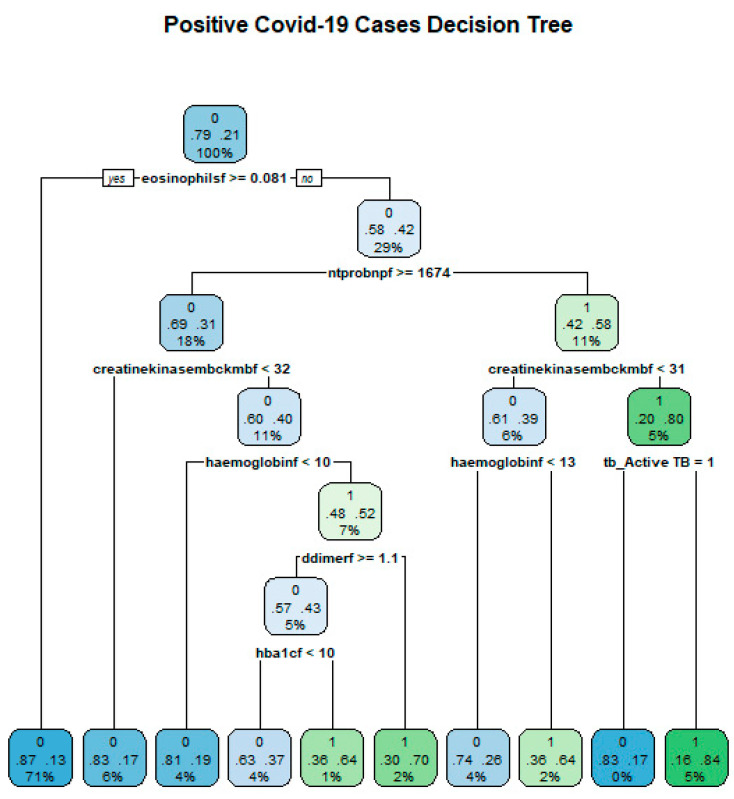
Decision Tree for risk stratification COVID-19.

Trees are characterised by two features, which are decision nodes and leaves. Leaves represent the decisions and/or the final label while decision nodes show points where data are divided. Figure 9 shows an example tree from the data and how classification can be conducted.

##### The Tree Building Algorithm

The algorithm commences by looking for a variable that divides the data into two nodes. This division is arrived at by minimising the impurity measurement at that node. A node that has two or more classes is impure while a node that has only one node is pure, hence a measure of impurity measures how much each node has multiple classes. The algorithm’s division is recursive and will continue until a certain stop criteria is achieved. Some examples of stop criteria include when a tree is too large and complex or when the set depth of the tree is reached [41].

##### Classification and Regression Tree Algorithm

For the Classification and Regression Tree (CART) algorithm, the impurity measure at each node is the MSE. This results in a tree, which is a collection of estimators at each node from the starting node to the terminal node [41]. In R, the study used the rpart package [42] to implement the CART algorithm.

##### Gini Importance

The Gini index is often used as a measure of impurity for splitting in tree-building algorithms for classification outcomes. The aim is to maximise the decrease in impurity at a node. A large Gini index indicates a large decrease in impurity at a node and hence a covariate split with a large Gini index can be considered to be important for classification [43,44]. Given that the decrease in impurity at a node, *h* is denoted as i(h), the Gini importance of a covariate, $X_{j}$ in a tree is the total decrease in impurity at all nodes of all trees in a forest ($I=\sum_{h}i\left(h\right)$) where the variable of interest is selected for splitting. That is the sum of all the Gini indices at all nodes in which covariate $X_{j}$ is selected for splitting. The average of all tree importance values for the covariate, $X_{j}$ is then termed the Gini importance of the random forest for $X_{j}$.

#### 3.4.2. Random Forest

A random forest is an ensemble of multiple CART decision trees [45]. The ensemble is fast and flexible, as it grows by bootstrapping without pruning the data. Random forest employs a modification of an algorithm known as bagging. They are many contemporary deployments of random forest algorithms but the most popular is Leo Breiman’s algorithm [40]. This involves a method of aggregating simple bootstrap to single tree general learners [40,46].

#### 3.4.3. Extension of the Bagging Algorithm

##### Out of Bag (OOB) Performance

RF are popularised because of their great OOB performance, usually giving high accuracy even from the default parameters in R (the study used the rfviz package [47]).

##### Variable Importance

Random forest models are usually considered black box, due to the ubiquity of the inner workings of the algorithm (Algorithm 2). To this end, for a degree of explainability it is recommended that one evaluate some form of variable importance when using RF models. Variable importance helps to obtain which covariates were more influential together with their degree of influence on the resulting classification model [48]. The most common measure of variable importance is usually the Gini importance and permutation importance, although the Gini importance is often biased. On the other hand, permutation importance bases its value on the effect of the covariate on the predictive power of the resulting forest [49].

#### 3.4.4. Extreme Gradient-Boosted Models (XGB)

**Figure 10:**
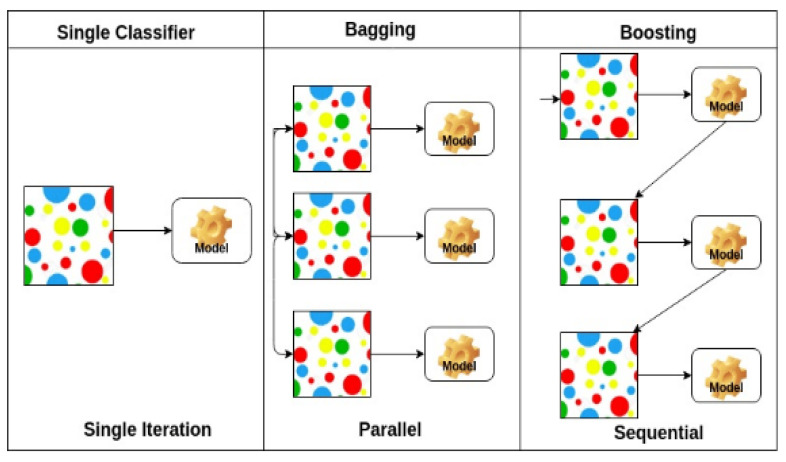
Illustration from Natekin and Knoll (2013) showing how the XGB model ensemble trees sequentially with improving on the previous ensemble as opposed to the bagging method used in RF and single classifiers such as LR and DT.

Extreme Gradient Boosting (XGB) is a family of ensemble of the same type of machine learning models [50]. For the data, the study used gradient boosting for classification; however, it was also applied in regression prediction. For extreme gradient boosting, they consist of an ensemble of decision trees. Unlike RF which ensemble deep independent trees (multiple trees connecting in parallel) XGB ensemble shallow trees (single trees connected in series). For this model, the study adds trees to the ensemble one tree per time [50,51]. Each tree seeks to correct and mend the errors that the previous ensemble model would have made [52]; see Figure 10 for illustration.
**Algorithm 2:** Random Forest Algorithm [44]Obtain training data (selecting a random number of data points) and select number of trees to be built (say *n* trees;For every tree, obtain a bootstrap sample and grow a CART tree to this data;For each bootstrap split, obtain *m* (where *m* is half the total number of all variables) variables out of all variables and select the best variable at the split. Then, divide the arising node into 2;Apply a tree stopping criteria (without pruning) to know when the tree is complete;From the above, obtain the output of this tree’s ensamblage.

With gradient boosting models, the study fitted models by the use of a differentiable loss function and an algorithm that minimises (optimises) gradient descent [52]. Presently, extreme gradient boosting is designed for high effectiveness and computational efficiency. This is because it uses an open-source approach to implement [53] and we used the xgboost package [54] in R.

### 3.5. Artificial Neural Networks (ANNs)

ANN are a mathematical copy of how the brain has an interconnected network of nodes called neurons. Each neuron is connected to another and can receive, process and output information. Neural networks are made of three main layers of neurons: (1) Input layer, (2) hidden layer and (3) output layer [55]. There are many arrangements and configurations onto which the various neurons connect to each other, and this configuration determines the type and how the network functions. A mathematical neuron has three main features [55]

1. Weighted inputs $\omega_{i}$;
2. An adder function that computes addition functions on the inputs;
3. An activation function that determines the type of the output.

Deep Learning, also known as Deep Structure Learning, is under the family of ANNs machine learning methods based on a group of algorithms based on a multi-layer NN that is able to perform various machine learning tasks [56]. These algorithms include more than one hidden layer in the NN structure, hence the name deep. The most common form of Deep Learning algorithms is the FFN, which allows information to move in the forward direction only without any recurrence [57]. Because of its computational flexibility, it is easy to tune hyper-parameters and good visual outputs, especially during the net training process; we used the Keras package in R [58] to fit both the SNN and the CNN.

#### 3.5.1. Self Normalising Neural Network (SNNs)

The study implemented the SNNs, a deep learning method capable of performing classification and regression (statistical models capable of predicting the values of an outcome *y*, using the values of predictor variables *x*) machine learning. Normalisation is changing and adjusting data values to a similar scale. The SNN network, unlike other NNs, uses a unique and different activation function called SELU as well as a unique and different dropout method, called the alpha dropout [59]. These two features provide the unique self normalising (normalising data without need of any human input) property of this neural network.

#### 3.5.2. Scaled Exponential Linear Units (SELU)

The SNNs use SELU as the activation function. SELU make the SNN self normalising by the construction of a special mapping *g*, which maps normalised inputs to outputs. RELU, leaky RELUs tanh unit and sigmoid units can not be used to construct SNNs [59]. SELU is defined, for any value, say *x*, as:

(5) $$SELU\left(x\right)=\lambda \left\{\begin{array}{cc} x, & \mathrm{if}\;x>0.\\ \alpha e^{x}-\alpha , & \mathrm{if}\;x\leq 0. \end{array}\right.$$

Hyperparameters, λ and α. λ>1, can be controlled to ensure that there are positive net inputs, which can be computed and normalised to obtain a Gaussian distribution. To make sure that the distribution mean is 0 and variance 1, set α=1.67326 and λ=1.0507.

##### Alpha Dropout

Another different feature that is presented by SNNs is their dropout technique. Dropout is defined as the method in which a NN ignores neuron units during the training phase. The ordinary dropout sets the activation of *x* to zero with probability (p), and hence keeps the average of the input distribution to the output distribution, albeit does not do so for the variance. For SNNs’ properties to be implemented, a NN needs to keep the mean and variance at 0 and 1, respectively. The standard traditional dropout fits RELUs (ReLU functions are either non-linear or linear but piece-wise, whose output equals the input for a positive input, otherwise the output is zero) and other activation functions without issues. Unlike the traditional dropouts, for SELU the *alpha dropout*, [59] is proposed, since the standard traditional dropout does not perform well with the SELU activation function. Hence, the alpha dropout is unique in two ways:

1. It randomly set the dropout input values to some value $\overset{´}{\alpha }$ instead of zero.
2. It keeps both the mean and variance at (0,1).

#### 3.5.3. Convolutional Neural Network

This study implemented a convolutional neural network (CNN) to the data. The CNN is a feed-forward neural network, with a depth of up to 30 layers and multiple cells. The layers are connected in a series (one after the other), with the hidden layers consisting of convolutional layers followed by either activation layers or pooling (poling layers are there to reduce the number of parameters and calculations required in the network). Convolutional layers are different from regular layers in other NNs because they use convolutions (convolutions sum two functions, usually polynomials, to obtain an output) rather than matrices [60,61]. Activation layers in CNN use different activation functions depending on the function of the network. This study used the sigmoid function, and RELU in the activation layers.

### 3.6. Measures of Model Performance

#### 3.6.1. Confusion Matrix

The study used the confusion matrix as given in Table 1 to define various measures of performance and thus compared how the models performed against each other (Visa et al., 2011).

#### 3.6.2. Accuracy, Precision, and Sensitivity

Accuracy, precision, and sensitivity are defined as [62,63]

$$\begin{array}{c} \mathrm{Accuracy}=\frac{\mathrm{TN}\;+\;\mathrm{TP}}{P\;+\;N}\\ \mathrm{Precision}=\frac{\mathrm{TP}}{\mathrm{TP}\;+\;\mathrm{FP}}\\ \mathrm{Sensitivity}=\frac{\mathrm{TP}}{\mathrm{TP}\;+\;\mathrm{FN}} \end{array}$$

Higher values of the accuracy, precision, and sensitivity demonstrate more correct predictions the model makes. Hence, a model with higher classification or regression accuracy, precision, and sensitivity will be better than that with lower accuracy, precision, and sensitivity [62,63].

#### 3.6.3. Cohen’s Kappa κ

This quantifies the reliability and accuracy of a classification method [64]. Unlike accuracy, Cohen’s κ takes into account the agreement that can happen merely by chance. Presently, it is defined as

$$\kappa =\frac{p_{0}-p_{e}}{1-p_{e}}$$

where $p_{0}$ is the relative observed agreement and $p_{e}$ is the chance agreement probability. Cohen’s κ ranges from 0 to 1 with κ=1, meaning that there is complete agreement, while with κ=0, there is no agreement.

#### 3.6.4. Predictive Values (PPV/NPV)

Predictive values measure the probability of correct predictions. Positive Predictive Value (PPV) is the probability that a model predicts a positive result given that the individual is actually positive. Negative Predictive Value (NPV) is the probability that a model predicts a negative result given the individual is actually negative [65]. The higher the values of PPV/NPV, the better the predictive performance of a model. Lower values of PPV/NPV indicates that the model predicts many false positives/negatives [65,66]. PPV and NPV are computed by:

$$\begin{array}{c} \mathrm{PPV}=\frac{\mathrm{TP}}{P^{\prime }}\\ \mathrm{NPV}=\frac{\mathrm{TN}}{N^{\prime }} \end{array}$$

#### 3.6.5. Receiver Operating Characteristic

The Receiver Operating Characteristic Curve (ROC) is a great way of visualising the performance of a classifier. The graph has been used for a long time to paint a picture of the trade-off between false alarm rates and hit rates of a classifier [67]. The ROC curve and Area Under Curve (AUC) have been mostly adopted in conjunction with other performance measures to provide a comprehensible comparison between classifiers [67,68]. TPR and FPR is defined as:

$$\begin{array}{ccc} \mathrm{FPR} & = & \frac{FP}{P+N}=\mathrm{Sensitivity}\\ \mathrm{TPR} & = & \frac{TP}{P+N}=\mathrm{Precision} \end{array}$$

The ROC graphs are plotted on a 2-D with TPR on the Y axis and FPR on the X-axis with Figure 2 and Figure 3 being examples of ROC curves. This thus shows the trade-off between gains (true positives) and losses (false positives) [68]. The higher the value of AUC, the better the performance of the model. An AUC value of 50% or less is worse than random guessing.

#### 3.6.6. The Wald Test

To test for the significance of a variable in a regression classifier, this study used the Wald test [69] at a 5% level of significance. Consider the LR model given in Equation (4) on Section 3.3:

$$\log\left(\frac{\pi_{i}}{1-\pi_{i}}\right)=\beta_{0}+\sum_{i=1}^{n}\beta_{i}\left(x_{i}\right)$$

The Wald test (sometimes known as the Z-test), tests the null hypothesis $H_{0}$:$\beta_{i}=0$ against an alternative hypothesis of $H_{1}$:$\beta_{i}\neq 0$. Failure to reject $H_{0}$ means that the variable whose coefficient is given by $\beta_{i}$ is not significant to the model, while rejecting $H_{0}$ means that there is enough evidence to suggest that the variable with coefficient $\beta_{i}$ is significant to the model [70].

## 4. Exploratory Data Analysis

This section describes various methods of data exploratory and steps that were taken to achieve the data structure as required by the analysis of the study. It also provides summary statistics and visualisations of the data.

### 4.1. Data Preparation

**Figure 11:**
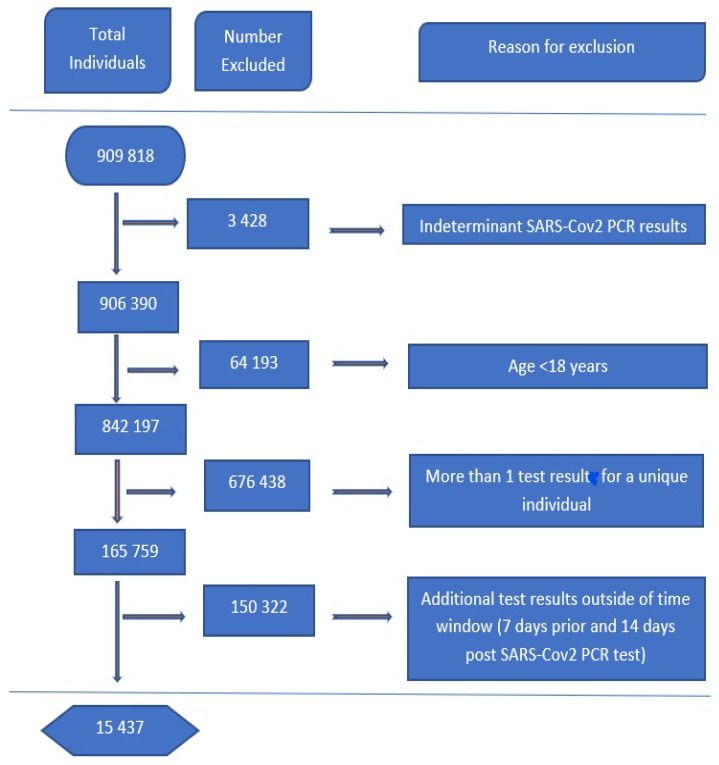
Data inclusion/exclusion.

For data preparation, we took all repeated test results from individuals who had two or more tests into one reading. An individual with at least one positive PCR COVID-19 test was labeled as positive. For other analytes and variables, an average over all the tests available was used. Analytes’ data was then filtered for time relevancy by taking results seven days prior and 14 days past the recorded COVID test result, although data concerning chronic diseases such as HIV, TB, and DM were taken six months preceding the SARS-CoV-2 test. The exclusion process is shown in Figure 11 and the summary of the demographics of the final data results are given in Table 2. For validation and re-sampling, the research split the data set into training and testing sets of the ratio 70:30, respectively. The data split was coupled together with 10-fold cross-validation repeated five times in each of the proposed models.

### 4.2. Missing Values and Imputation

**Figure 12:**
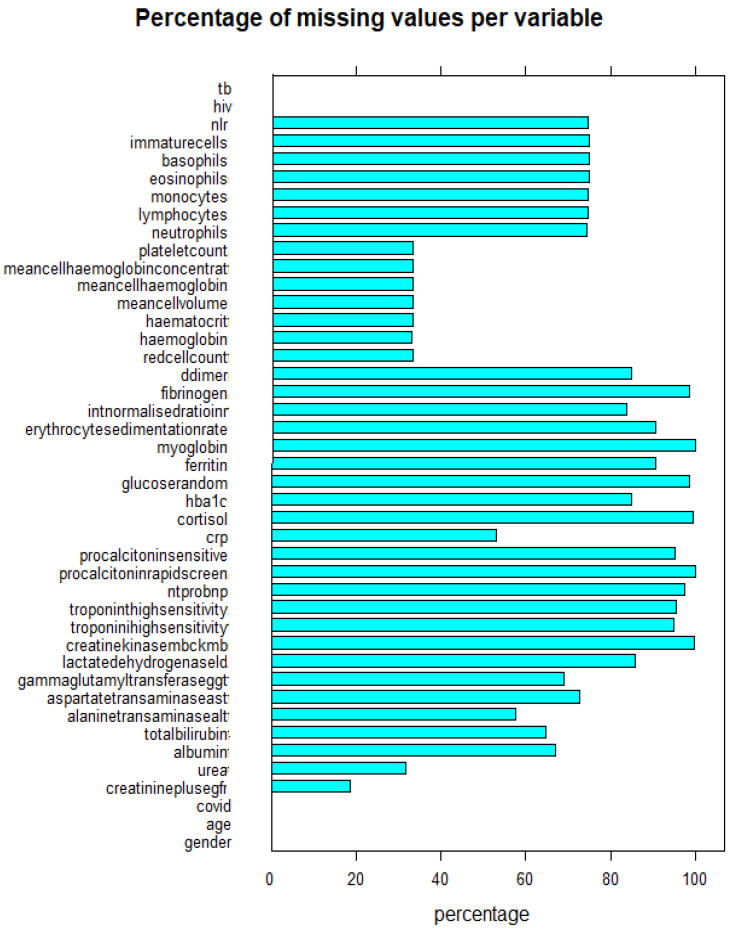
Percentage of missing values in the data per variable.

Figure 12 (the blue bars represent the percentage of missing values for each variable), shows that most variables have at least 80% missing values. The missingness in the data was structural because not all tested patients were admitted to obtain their routine blood tests. To deal with missing values, we used the missForest package in R [21], which is robust to deal with such an amount of missing values, and compared it with simple statistics missing values imputation (SSMVI) using measures of central tendency, specifically the mean and mode. Results of the comparison of the methods coupled with two robust variable selection methods, applied on the base ML method of logistic regression are shown on Table 3.

### 4.3. Variables and Variable Selection

#### 4.3.1. Variables

The data contained analytes that were grouped by physiological system as follows: inflammatory [Creactive protein (CRP), IL-6, procalcitonin (PCT), ferritin, erythrocyte sedimentation rate (ESR)], coagulation (D-dimer; INR; fibrinogen), full blood count [white cell count (WCC) total, red cell count, haemoglobin, haematocrit, mean corpuscular volume, mean corpuscular haemoglobin, mean corpuscular haemoglobin concentration, red cell distribution width, platelet count], WCC differential [absolute count, neutrophil, lymphocyte, monocyte, eosinophil, basophil, as well as the neutrophil to lymphocyte ratio (NLR)], liver related [aspartate aminotransferase (AST), alanine aminotransferase (ALT), gamma-glutamyl transferase (GGT), lactate degydrogenase (LDH), total bilirubin, albumin)], cardiac related [troponin T, troponin I, N-terminal pro b-type natriuretic peptide (NT-proBNP)], endocrine related (HbA1c) and renal function-related [urea, creatinine, estimated glomerular filtration rate (eGFR)].

#### 4.3.2. Variable Selection

To begin with, the research removed features that had a confounding effect on the results and/or those that were created because of a positive COVID-19 test exemplified by features such as severity of the COVID-19 disease and some which included results of the methods that are used to arrive at HIV, TB and DM results. Eventually, there was a total of 37 variables with names displayed in Table 4.

We compared the two methods of variable selection, namely the LASSO and the Boruta algorithm. The two methods are paired with the two methods of missing data imputation and the resulting data was run through the LR base model. The results of the comparative permutations of the methods of missing value imputation and variable selection applied on our base ML model are given in Table 3.

In Table 3, the top performing measure is coloured in red. The combination of the missForest missing values imputation method and the Boruta variable selection algorithm outperforms the other three permutations on five out of seven measures of the model performance. Thus, we concluded that both missForest missing value method and the Boruta algorithm for variable selection were the best methods for the data given. The data and variables selected from the combination of the two methods is used in further ML modelling both in status prediction and risk stratification.

#### 4.3.3. Boruta Variable Selection

**Figure 13:**
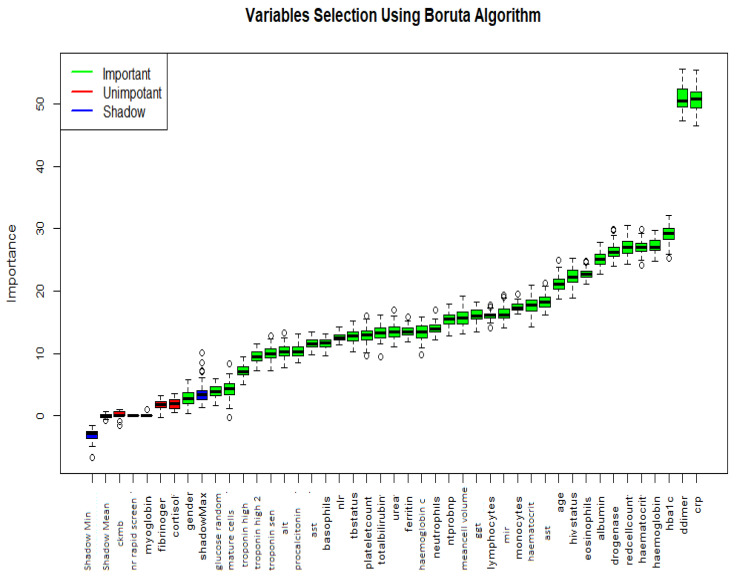
Variable selection using the Boruta Algorithm. Note that 5 variables were deemed less useful with the other 38 deemed useful.

We ran the initial 43 variables and data obtained after missing value imputation using missForest in the Boruta algorithm. The results of the variable selection are shown in the Figure 13. The variables with green (38 variables) shaded box plots are the important variables, with those in red (five variables) not being of importance to the model. Shadow variables are shaded in blue. The variables whose box-plots are coloured in blue are selected for use in this study.

## 5. Results: Risk Stratification

This section analyses results of ML models fitted for risk stratification (RS). As noted, a critical case was defined as a patient who was admitted into a ward because of COVID-19 complications, non critical patients are positive cases that were not admitted. Of the 3301 positive cases, 1036 were classified as risky and 2265 not risky. The study fitted each model with a 70% training set and 30% test set, together with 10-fold cross validation repeated 5 times for re-sampling.

### 5.1. Logistic Regression

**Figure 14:**
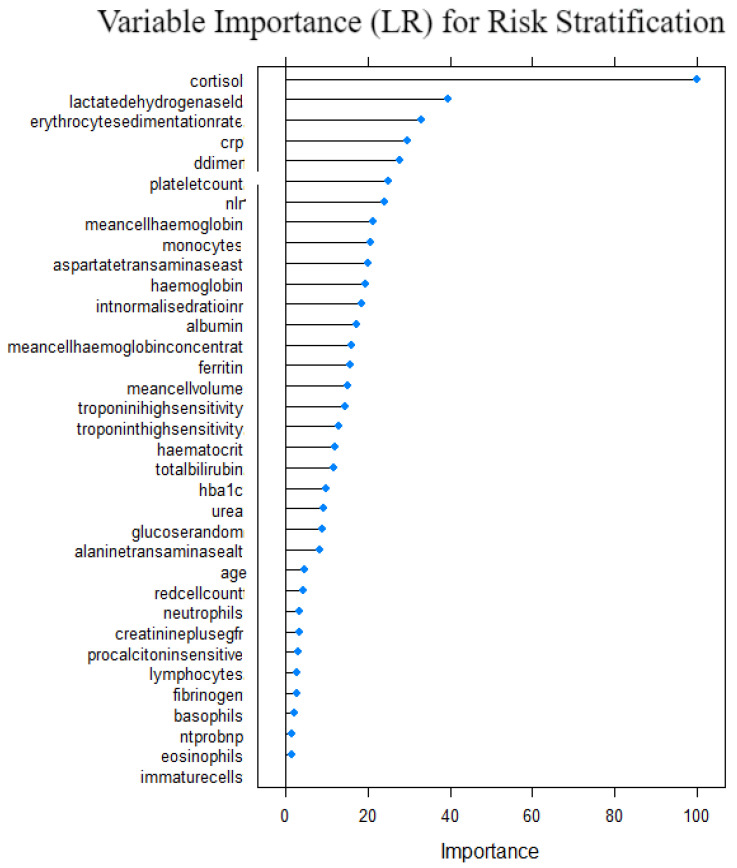
Variable importance from LR on risk stratification.

The study fitted the Equation given on Section 3.6.6 with the predicted variable being severity of the patient, i.e, critical or not critical and the predictors as the variables selected from running the Boruta algorithm as given in Section 4.3.3. Table 5 shows the values of the coefficients $\beta_{i}$ and intercept $\beta_{0}$ for all variables *i* in the fitted model. Wald tests for variable significance at 5% level of significance and the null hypothesis $H_{0}$:$\beta_{i}=0$ against an alternative hypothesis of $H_{1}$:$\beta_{i}\neq 0$. Figure 14 shows variables that were important to the logistic regression in risk stratification. Cortisol, lactate dehydrogenase, erythrocyte sedimentation rate, crp and d-dimer were the top five important variables whilst, immaturecells, eonophils, ntprobnp, basophil and fibrinogen were the five least important variables to the model. This agrees with the Wald test results. LR model being the base model was used to compare with others, as it outperforms DT; however, it did not outperform the other five models. The results of LR model performance compared to other models are given in Table 6 and Section 5.7.

### 5.2. Decision Tree

**Figure 15:**
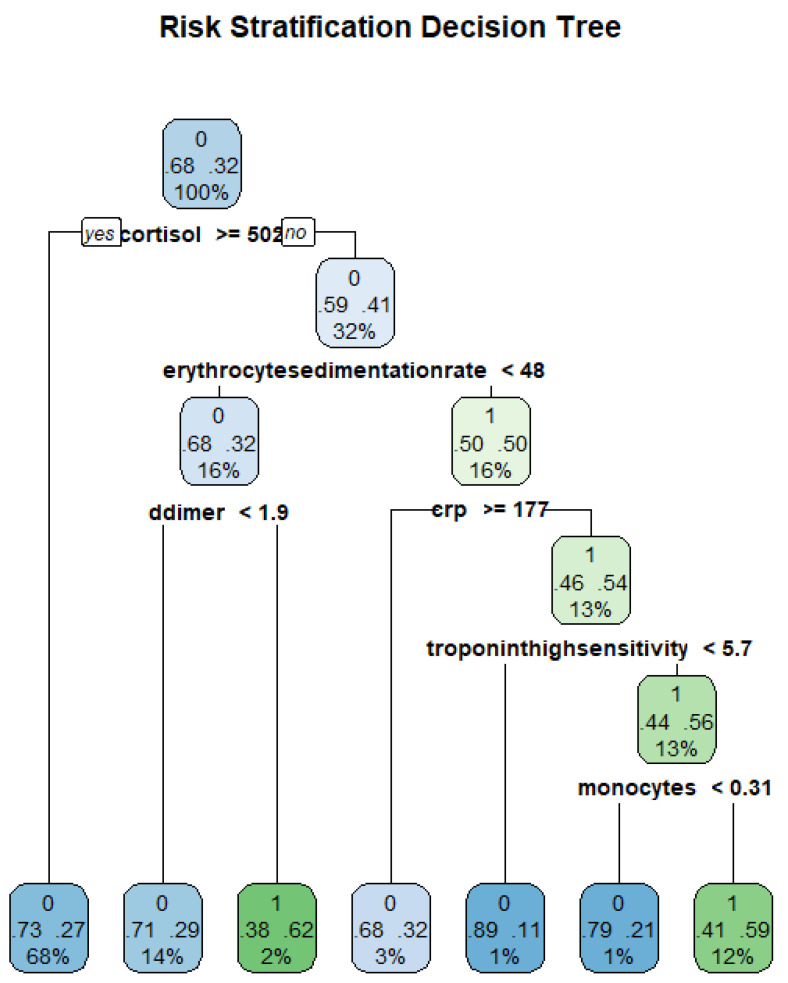
Decision tree structure for COVID-19 risk stratification.

The study fitted a regression tree using the Gini index as the impurity measure at each node. Figure 15 shows the resulting DT from the data.

**Figure 16:**
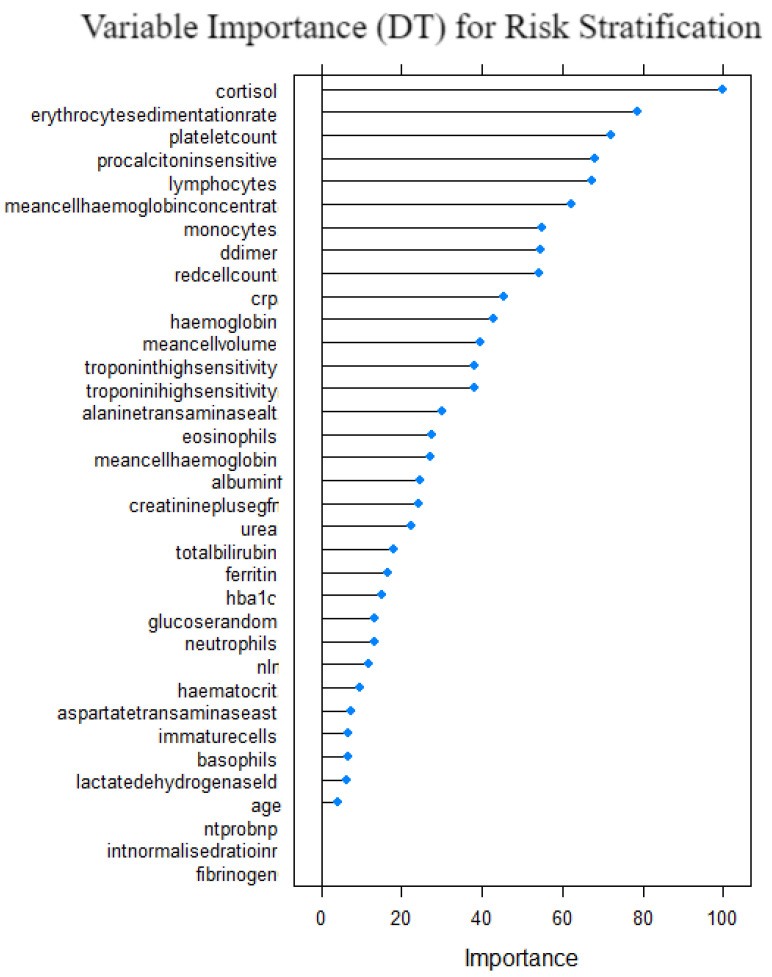
Variable importance for decision tree model on risk stratification.

Figure 16 shows the variable importance for the decision tree model. Cortisol, erythrocytesedimentation, plateletcount, procalcitonin and lymphocytes were the variables with top 5 importance measure. Fibrinogen, INR, nt prob, age and LDH were the least important variables. This suggests that they are significant to the risk stratification of COVID-19 patients when the decision tree model is used. The computed measures of model performance for the decision tree model are given in Table 6 and Section 5.7, which demonstrates that although the model performed well in absolute terms, it was the fourth performing model relative to the other six models. It outperformed the base model LR and XGB model.

### 5.3. Random Forest

**Figure 17:**
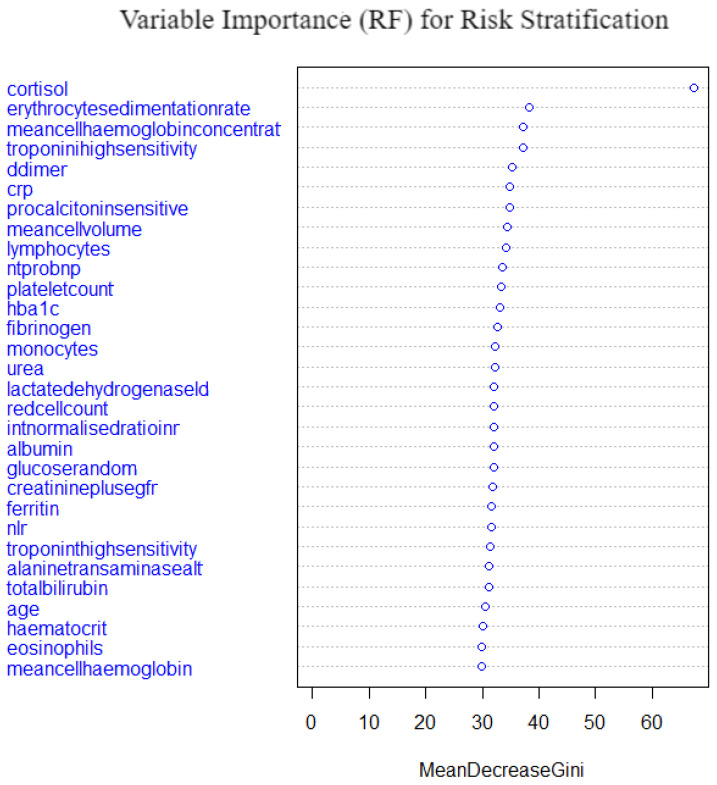
Variable importance from random forest model on risk stratification.

A random forest of 500 trees with 6 variables being tried at each split was fitted. For resampling, a 10-fold cross validation method was employed and the OOB error was low at 18.29%, which demonstrates a high performance of the model’s predictive power. Figure 17 shows the variable importance from the fitted model. Cortisol, erythrocyte sedimentationrate, mean cell haemoglobin concentration, troponin high sensitivity, d-dimer and crp were the top important variables. Meancellhaemoglobin, eosinophils, haematocrif, age and totalbilirubin are the least important variables. Cortisol shows an irregularly higher importance compared to the other important variables; this may be because it is a stress hormone. The model performance as compared to the other models is shown in Table 6 and Section 5.7. The random forest model is the second best performing model overall after the self normalising neural network.

### 5.4. Extreme Gradient Boosting

**Figure 18:**
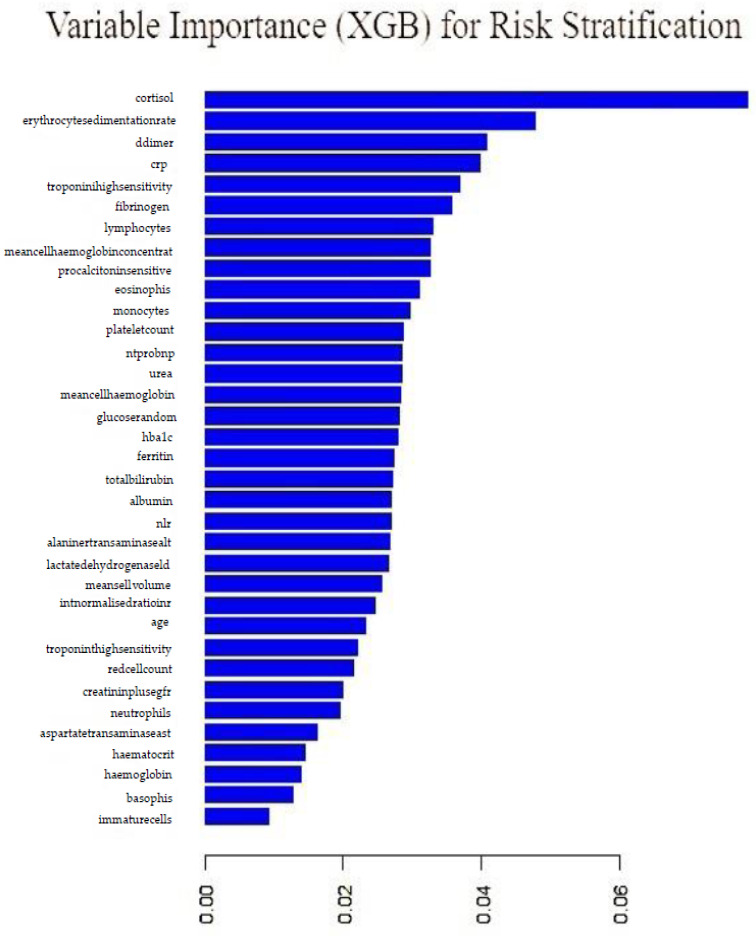
Variable importance from extreme gradient boosting model.

An extreme gradient boosting model employing stochastic gradient boosting with 120 iterations, with the final model employing 150 trees, was fitted. The final model with 150 trees and accuracy of 70% and a kappa value of 18% was employed. Figure 18 shows variable importance from the gradient boosting model. Cortisol, erythrocyte sedimentation rate, d-dimer, crp and troponin were the top five important variables. The least performing variables were: immature cells, basophils, haemoglobin, haematocrit and aspartatetransaminase.

### 5.5. Convolutional Neural Network

A deep feed-forward convolutional neural network (CNN) with one hidden layer was fitted to the data for risk stratification. The input layer had 256 neurons with RELU activation function and a dropout rate of 0.4, the hidden layer had 128 neurons with RELU activation function and 0.3 drop out rate. The output layer had a sigmoid activation, as it works best for binary outputs. Binary cross-entropy was the loss function employed as well as using the ADAM learning rate optimiser. Table 6 shows that the convolutional neural network was the third best performing model after the random forest model and self normalising neural network. **Note:** that there is no variable importance for this neural network model due to its black box nature, as well as the keras package does not have the feature.

### 5.6. Self Normalising Neural Network

The study fitted a deep feed forward self-normalising neural network (SNN) with two hidden layers to the data for risk stratification. The input layer had 128 neurons with SELU activation function and a dropout rate of 0.4, the first hidden layer had 64 neurons with SELU activation function and 0.3 drop out rate. The second hidden layer had 32 neurons, SELU activation and dropout rate of 0.1. The output layer had a sigmoid activation, as it works best for binary outputs. Binary cross-entropy was the loss function employed as well as using the ADAM learning rate optimiser. Table 6 shows that the self normalising neural network was the second best performing model after the random forest model. **Note:** that there is no variable importance for this neural network model due to its black box nature, as well as the fact that the keras package does not have this feature.

### 5.7. Model Performance Comparison and Discussion

**Figure 19:**
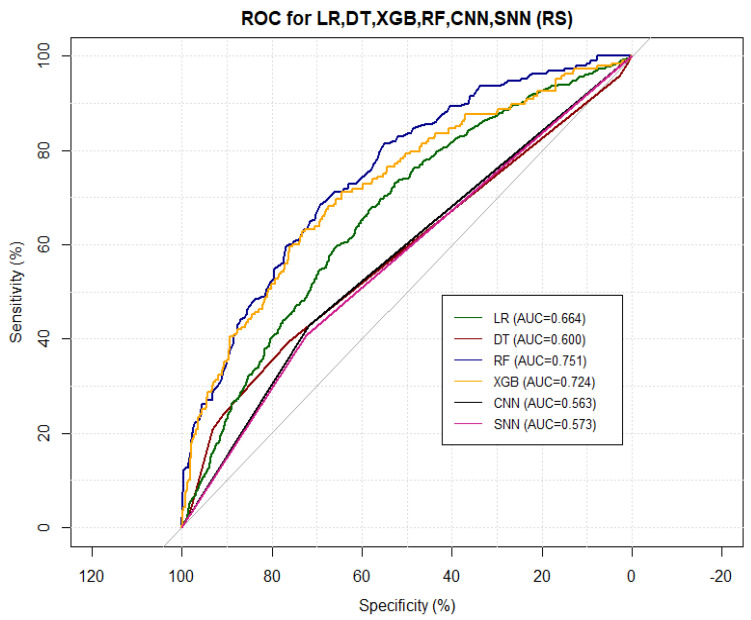
Combined ROC curves.

Table 6 shows how the different models performed in COVID-19 risk stratification. The top performing model value for each measure of performance is coloured in red whilst the second best value is coloured in green. Figure 19 shows the corresponding ROC curves of the models. From Table 6 and Figure 19, here is the average rank of the models’ performance from the best to the worst:

1. Self normalising neural network;
2. Random forest;
3. Convolutional neural network;
4. Decision tree;
5. Extreme gradient boosting;
6. Logistic regression.

These results are consistent with existing literature, as summarised by [23]. Table 7 shows the important variables from the four models we fitted and the ones reported by over 20% of other research on the same subject, as summarised by [23] and given in Figure 7. All the models’ important variables are the same as those reported in literature, the exceptions being age, Oxygen and blood urea nitrogen and comorbidity, which were reported by the other studies but were not used in this study. **Note:** that there is no variable importance for neural network models due to their black box nature, as well as the fact that the keras package does not have the feature.

### 5.8. Conclusions

The models fitted in this research and the results obtained are consistent with the ones presented in the existing theory, summarised by [23]. However, not many studies cited used deep learning models. The base model (LR) performed very well, as did the other five models fitted. Below, we present the ranking of the models’ overall performance on risk stratification:

1. Self normalising neural network;
2. Random forest;
3. Convolutional neural network;
4. Decision tree;
5. Extreme gradient boosting;
6. Logistic regression.

The reported variables that were important to various models are also consistent with the literature, as summarised by [23] and shown on Table 7 and Figure 7. It is noted that cortisol was recorded as important by all models fitted in this study but not by more than 20% of other studies in literature. We also noted that oxygen is recorded in literature as a big risk factor by other studies in literature but not by any of the models fitted in this study. The data used in this study did not contain oxygen saturation. These two deviations from the literature calls for further study.

## 6. Discussion and Conclusions

We proposed the use of six machine learning algorithms (LR, DT, RF, XGB, CNN and SNN) to predict COVID-19 risk stratification using routine lab blood tests, other comorbidities such as HIV, TB and DM as well as a few demographic features of individuals. The chosen methods of approach had high perfomance.

SNN was the best and RF was the second best performing model. All models performed better than the base model. The highest AUC and sensitivity were 75% and 98%, respectively, both recorded from the RF model, whilst the top specificity and NPV were 68% and 81%, respectively, with both recorded from CNN. SNN recorded the highest PPV and accuracy, with values of 76% and 75%, respectively. DT performed better than all models on the Kohen’s kappa, with a recorded value of 32%.

Cortisol, commonly known as the stress hormone, is the top important variable in four of the models implemented. This is expected, as the body will produce more cortisol when under stress from the virus. Erythrocyte sedimentation rate is a variable that shows the rate of red blood cells sedimentation, which measures the rate of body inflammation. The body is expected to have a high rate of the sedimentation due to stress from the virus. LDH, CRP, D-dimer and fibrinogen are also top variables common in all the model’s variable importance. These top variables are also capable of capturing the clinical pathway/trajectory, as they are vital signs of the disease. They are dynamic and can be used for better predictive utility during the time of hospitalisation of hospitalised patients.

Unlike earlier studies in literature, which proposed machine learning models on CT images for risk stratification, using routine laboratory tests data is economically and computationally cheaper, as well as faster.

The models that we implement in this study were used by various other researchers on the same topic. These studies are gathered up together and summarised by [23], and Figure 6 shows the commonly used models in these studies. Although few studies use neural networks, just a handful, if any, used CNN and SNN. The model performance results agree with the literature as well.

Table 7 show the features/variables that were important to models used in this study. The results as shown in the tables are in concordance with the literature. The important variables from our models such as: age, LDH and d-dimer were also presented in more than 20% of the studies.

### 6.1. Strengths

- The use of data from NHLS gives a sample with a fair representation of the population, because most studies use hospital centralised data rather than nation-wide data.
- The use of over 30 laboratory blood test analytes, comorbidities and demographics, gives a variety of prediction variables. Other studies focus on only one family of variables, i.e, either focusing on blood test analytes or comorbidities or demographics.
- The use of six ML models with dissimilar approaches gives a wide range of techniques to choose from and is useful for comparative purposes. Most studies employed 2 or 3 models.

### 6.2. Limitations and Improvements

- The data had a lot of missing values, with some variables having at least 80% missing values, and with less missing values the ML models could perform better than they did.
- The data was not balanced. To balance the data, the study used a data balancing method that replicate the data. The method was not efficient.
- Over-fitting during the preliminary results was a potential issue, but we solved this by balancing the data as well as normalising some variables. Cross-validation, as well, helped mitigate this issue as it was employed for all the models given.
- The data did not have information on oxygen saturation, which was demonstrated to be important in other studies in literature, and the addition of a simple observation such as oxygen saturation may improve the model significantly.
- We could improve intepretability by using SHapley Additive Explanation (SHAP) and Local Interpretable Model Agnostic Explanations (LIME) frameworks.

### 6.3. Clinical Integration

The proposed algorithms, particularly the top two performing models—RF and SNN, can be integrated into most of the clinical support software with the aim of identifying affected patients and risk stratification. Sensitivity and specificity on prediction for RF are 96.25% and 57.76%, respectively, whilst the same is 85% and 86.29%, respectively for SNN. Sensitivity and specificity on risk stratification for RF are 98.44% and 15.43%, respectively, whilst the same is 65.32% and 65.82%, respectively for SNN. The risk of the integration of the algorithms lies more on false positives and false negatives. This is because of the need to limit false positives, as they can take up space for the patients who need care, whilst avoiding false negatives for patients in need of hospital care. The predictive performances shown by the top two algorithms (RF and SNN) are high specifically on prediction, and hence we can minimise the risks.

This study demonstrated that we can feasibly use machine learning models to stratify the risk associated with COVID-19, using routine laboratory test data, demographics and comorbidities. The robust and high performance results from the proposed models was confirmed on test and validation data sets for all the models. RF and SNN models were top performing models and the other four fitted models’ performance were not far off either. The study has demonstrated greater agreement with existing literature, on the variables that are important to COVID-19 risk stratification. Thus, the proposed models and methods can be integrated into hospital and clinical systems for rapid patient identification and risk stratification. This is particularly important in the remote parts of South Africa and Africa at large where there might not be a laboratory capable of performing RT-PCR.

## Figures and Tables

**Table 1:** Confusion matrix.

	Model Predicted Values			
**Actual Values**		Positive	Negative	Total
	Positive	True positive (TP)	False Negative (FN)	P${}^{\prime }$ = TP + FN
	Negative	False Positive (FP)	True Negative (TN)	N${}^{\prime }$ = FP + TN
		P = TP + FP	N = FN + TN	T = P + N = P${}^{\prime }$ + N${}^{\prime }$

**Table 2:** Basic demographics.

Demographic Variables	Total Number	Positive	Negative
Gender			
Male	6513	1320	5193
Female	8768	1958	6810
Other/unknown	156	23	133
Age groups			
18–39 years	5839	870	4969
40–60 years	5771	1385	4386
Above 60 years	3827	1046	2781
HIV status			
Positive	4055	502	3553
Negative/unknown	11,382	2799	8583
TB status			
Positive	863	59	804
Negative/Unknown	14,574	3242	11,332

**Table 3:** Comparative permutations of the methods of missing values imputation and variable selection applied on the base ML model (LR).

	Simple Statistics Imputation		missForest Imputation	
	Boruta Algorithm	LASSO Algorithm	Boruta Algorithm	LASSO Algorithm
Accuracy	80.31	79.53	84.42	75.10
Kappa	19.02	11.87	47.04	34.64
Sensitivity	97.80	99.09	46.12	34.64
Specificity	15.96	9.02	94.90	84.76
PPV	81.06	79.70	71.23	39.29
NPV	66.39	73.33	86.54	84.00
AUC	63.29	70.80	86.00	61.30

**Table 4:** List of variables used.

**Demographics**	**Kidney Function Tests**	**Liver Function Tests**
age	Creatinine	Albumin total (ii) bilirubin
Gender (M = M, F = F)	eGFR	Alanine transaminase (alt)
HIV	Urea	Lactatede hydrogenase (ld)
TB		Aspartate transaminase (ast)
		Gamma glutamyl transferase (ggt)
**Diabetes markers**	**Haematology**	**Neutrophil to lymphocyte ratio** **Coagulation markers**
Glucose random	Ferritin	International normalized ratio
hba1c (hemoglobin A1c)	Erythrocyte sedimentation rate	(ii) fibrinogen
	Myogloin	(iii) d-dimer
	Monocytef eosinophils basophils	
	D-dimer redcellcount	
	Haemoglobin haematocrit	
	Meancellvolume	
	Meancell haemoglobin concentrat	
	Immaturecells	
	Meancellhaemoglobin	
**Cardiac biomarkers**	**Inflammatory markers**	
Creatine kinase(ckmb)	Neutrophils	
Troponin high sensitivity	Procalcitonin sensitive	
ntprobnp	Crp (c-reactive protein)	
	Cortisol	
	Procalcitonin rapid screen	

**Table 5:** Summary of the results of the logistic regression model for risk stratification.

Variable	Coefficient $\beta_{i}$	Significance
Intercept	7.77 × 10${}^{0}$	
Age	−1.433 × 10${}^{-3}$	sig
CreatinineeGFR	1.426 × 10${}^{-4}$	not sig
Urea	−6.104 × 10${}^{-3}$	not sig
Albumin	−1.986 × 10${}^{-2}$	sig
Totalbilirubin	3.646 × 10${}^{-3}$	sig
Alanine transaminase	−1.095 × 10${}^{-3}$	not sig
Aspartate transaminase	2.121 × 10${}^{-3}$	sig
Lactate dehydrogenase	−1.238 × 10${}^{-3}$	sig
Creatine kinase	6.906 × 10${}^{-2}$	sig
Troponin highsensitivity	5.627 × 10${}^{-4}$	not sig
Nt probn	1.504 × 10${}^{-6}$	not sig
Neutrophils	−7.154 × 10${}^{-3}$	not sig
Procalciton insensitive	−8.988 × 10${}^{-4}$	sig
Crp	1.919 × 10${}^{-3}$	sig
Cortisol	−4.539 × 10${}^{-3}$	sig
hba1c	2.472 × 10${}^{-2}$	not sig
Glucose random	−8.645 × 10${}^{-3}$	not sig
Ferritin	1.026 × 10${}^{-4}$	not sig
Erythrocyte sedimentation rate	1.058 × 10${}^{-2}$	sig
Int normalised ratio inr	2.823 × 10${}^{-1}$	sig
Fibrinogen	2.527 × 10${}^{-2}$	not sig
D dimer	5.455 × 10${}^{-2}$	sig
Redcellcount	2.304 × 10${}^{-1}$	not sig
Haemoglobin	−4.404 × 10${}^{-1}$	sig
Haematocrit	1.055 × 10${}^{1}$	not sig
Mean cell volume	−8.338 × 10${}^{-2}$	not sig
Mean cell haemoglobin	3.541 × 10${}^{-1}$	sig
Mean cell haemoglobin concentration	−2.521 × 10${}^{-1}$	not sig
Platelete count	1.085 × 10${}^{-3}$	sig
Monocytes	−3.767 × 10${}^{-1}$	sig
Eosinophils	−7.946 × 10${}^{-2}$	not sig
Basophils	4.290 × 10${}^{-1}$	not sig
Immature cells	−2.843 × 10${}^{-2}$	not sig
Nleutrophil lymphocyte ratio	2.945 × 10${}^{-2}$	sig
Gender_M	1.112 × 10${}^{0}$	not sig
HIV positive	−3.8956 × 10${}^{-2}$	sig
TB positive	2.330 × 10${}^{0}$	sig

**Table 6:** Model performance comparison for risk stratification.

	LR	DT	RF	XGB	CNN	SNN
AUC	66.41	60.00	75.11	72.37	56.53	57.31
Sensitivity	94.88	93.45	98.44	94.21	63.43	65.32
Specificity	11.20	34.55	15.43	27.66	68.41	65.82
PPV	69.59	75.36	73.54	75.67	74.22	75.78
NPV	50.53	71.12	80.56	66.67	81.26	71.30
Accuracy	68.24	74.70	73.94	74.57	70.23	75.28
Kappa	07.67	32.45	18.13	26.35	26.79	31.14

**Table 7:** Top important variables in COVID-19 risk stratification.

LR	DT	RF	XGB	>20% Studies
Cortisol	Cortisol	Cortisol	Cortisol	Age
LDH	Erythrocyte sedimentation rate	Erythrocyte sedimentation rate	Erythrocyte sedimentation rate	LDH
Erythrocyte sedimentation rate	Platelet count	Mean cell haemoglobin concentration	CRP	Lymphocytes
CRP	Procalciton insensitivity	Troponin insensitive	Troponin insensitive	CRP
D-dimer	Lymphocytes	D-dimer	Fibrinogen	Oxygen
Platelet count	Mean cell haemoglobin concentration	CRP	Lymphocytes	BUN
nlr	Monocytes	Mean cell haemoglobin concentration	Procalciton insensitive	D-dimer
Mean cell haemoglobin concentration	D-dimer	Lymphocytes	Procalciton insensitive	Comorbidities

## Data Availability

The data used in this study is available upon application from the Central Data Warehouse (CDW) of the National Laboratory Health Services (NHLS).
